# Phosphate-Solubilizing Bacteria Nullify the Antagonistic Effect of Soil Calcification on Bioavailability of Phosphorus in Alkaline Soils

**DOI:** 10.1038/s41598-017-16537-5

**Published:** 2017-11-23

**Authors:** Muhammad Adnan, Zahir Shah, Shah Fahad, Muhamamd Arif, Mukhtar Alam, Imtiaz Ali Khan, Ishaq Ahmad Mian, Abdul Basir, Hidayat Ullah, Muhammad Arshad, Inayat-Ur Rahman, Shah Saud, Muhammad Zahid Ihsan, Yousaf Jamal, Amanullah, Hafiz Mohkum Hammad, Wajid Nasim

**Affiliations:** 1 Department of Agriculture, The University of Swabi, Swabi, Pakistan; 2 Department of Soil and Environmental Sciences, the University of Agriculture, Peshawar, Pakistan; 3 College of Plant Science and Technology, Huazhong Agricultural University, Wuhan, China; 4 Department of Agronomy, the University of Agriculture, Peshawar, Pakistan; 5 Mountain Agriculture Research Center Gilgit Bultistan, Bultistan, Pakistan; 6 College of Horticulture, Northeast Agricultural University, Harbin, China; 7 Cholistan Institute of Desert Studied, The Islamia University of Bahawalpur, Bahawalpur, Pakistan; 8 Deparment of Agronomy, the Islamia University of Bahawalpur, Bahawalpur, Pakistan; 9 Department of Environmental Sciences, COMSATS Institute of Information Technology, Vehari, 61100 Pakistan; 10 CIHEAM-Institute Agronomique Mediterraneen de Montpellier (IAMM), Montpellier, 34090 France; 11 CSIRO Ecosystems Sciences and Sustainable Agriculture Flagship, Toowoomba, QLD 4350 Australia

## Abstract

Phosphate-solubilizing bacteria (PSB) reduce the negative effects of soil calcification on soil phosphorus (P) nutrition. In this incubation study, we explored the ability of PSB (control and inoculated) to release P from different P sources [single super phosphate (SSP), rock phosphate (RP), poultry manure (PM) and farm yard manure (FYM)] with various soil lime contents (4.78, 10, 15 and 20%) in alkaline soil. PSB inoculation progressively enriched Olsen extractable P from all sources compared to the control over the course of 56 days; however, this increase was greater from organic sources (PM and FYM) than from mineral P sources (SSP and RP). Lime addition to the soil decreased bioavailable P, but this effect was largely neutralized by PSB inoculation. PSB were the most viable in soil inoculated with PSB and amended with organic sources, while lime addition decreased PSB survival. Our findings imply that PSB inoculation can counteract the antagonistic effect of soil calcification on bioavailable P when it is applied using both mineral and organic sources, although organic sources support this process more efficiently than do mineral P sources. Therefore, PSB inoculation combined with organic manure application is one of the best options for improving soil P nutrition.

## Introduction

Phosphorus (P) is an essential plant nutrient and plays a key role in plant growth and development. It is the world’s second largest nutritional supplement for crops after nitrogen. On average, soil contains 400–1000 mg kg^−1^ of total P, of which only 1.00–2.50% is available to plants for uptake^1^. Phosphorus is found in soil in both mineral and organic forms. Most of the organic P (20 to 80%) has been found to be inert^2^. Mineral P in the soil may become unavailable through fixation or adsorption in clay soil^3^. Mineral P may also become unavailable because of precipitation reactions with cations such as Ca-P and Mg-P in alkaline soil or Fe-P and Al-P in acidic soil^4,5^. As a result, the concentration of mineral P in the soil solution rarely exceeds 0.1 mg kg^−1^.

Calcareous soils, such as Inceptisols, Entisols, Alfisols and Vertisols, are most abundant in arid and semi-arid zones of the world. In Pakistan, most of the soils are calcareous in nature, and consequently, 90% of soils in Pakistan are moderately to severely deficient in phosphorus^6^. In Pakistan, the total application of P fertilizer (DAP and TSP) for wheat cultivation alone is estimated to be 250,500 tons, although only 15–20% of the applied P becomes available to plants^7^. Globally approximately 30 million tons of P fertilizers (DAP and TSP) are used every year with an actual uptake efficiency of only 20% almost 80% of the applied P goes out of the soil plant system^8^. On other hand, organic manure and compost application to calcareous soils may form Ca–organic P complexes, such as Ca-phytates, which may affect soil P chemistry^9^. P applied to calcareous soils through mineral fertilizers is fixed soon and most of it becomes unavailable. A substantial amount precipitates as [Ca_3_(PO_4_)^2^] and [Mg_3_(PO_4_)^2^]^10^. This indicates that lime usually used as calcite, is a chief sorbent of orthophosphate in calcareous soils^11^. Consequently the farmers are compelled to use of P fertilizer recurrently at a huge cost and labour^12^. P as mineral fertilizer is costly and an environmentally detrimental practice.

Phosphorous (P) uptake by crop can be improved by enhancing P solubility in soil solution and/or decreasing P fixation in soil. The unavailable P compounds can be made available for the plant by phosphate solubilizing bacteria (PSB). The active strains of PSB involved in this conversion are Pseudomonas, Mycobacterium, Micrococcus, Bacillus, Flavobacterium, Rhizobium, Mesorhizobium and Sinorhizobium^13^. PSB have been reported to modify P nutrition and increase its solubalization in soil through many process such as, they may decrease the pH of the soil by the producing organic (gluconic acid) and mineral acids^14^, alkaline phosphatases^15^, phytohormones and H+ protonation^16^, anion exchange, chelation and siderophores production which promote P solubilization in soil^17^. Exploitation of these processes may prevent frequent addition of P into soil with a substantial reduction in cost of production to the farmers and damage to the environment^18^. Zaida *et al*.^19^ have previously reported significant improvement in P availability for plants through PSB inoculation. The use of PSB promote the growth of plant in other way such as by speeding up seed germination, improving seedling emergence, increasing resistance to abiotic stress, guard plants from disease and improving root morphology^20^.

Due to a high concentration of calcium in the soil and an alkaline pH, phosphorous availability is critical in calcareous soils. The viability of phosphate-solubilizing bacteria (PSB) as well as their potential to improve P nutrition may vary depending upon soil, climatic conditions, and P source (i.e., organic, natural, and synthetic). Accordingly, this incubation experiment was performed to evaluate the role of PSB in improving P solubilization/mineralization from organic and mineral P sources in artificially amended calcareous soils.

## Results

### PGPR characteristics of PSB inoculum

We observed substantial plant-growth-promoting rhizobacterial (PGPR) characteristics in the PSB used in the experiment (Table 1). The inoculated bacteria were capable of solubilizing phosphate (8.5 ± 0.53 diameter of halo in mm) and produced siderophores (5.7 ± 0.81 diameter of halo in mm), IAA (8.4 ± 0.61 µg ml^−1^), auxin (3.7 ± 0.42 mg ml^−1^), and organic acids (10.7 ± 0.73 g L^−1^).

**Table 1:** Indole acetic acid (IAA), phosphate- solubilization, organic acid, siderophore and auxine production by applied PSB inoculum.

Phosphate-solubilization (diameter of halo in mm)	8.5 ± 0.53
Production of siderophores (diameter of halo in mm)	5.7 ± 0.81
IAA production (µg ml^−1^)	8.4 ± 0.61
Auxin production (mg ml^−1^)	3.7 ± 0.42
Total organic acid (g L^−1^)	10.7 ± 0.73

Values represent the mean of 3 replications.

### Phosphorus release in relation to PSB inoculation, P sources, soil calcification and their interactions

PSB inoculation significantly (P ≤ 0.05) improved P release over the uninoculated control at all incubation intervals (7, 14, 28 and 56 days) except day zero (Tables 2 and 3). Olsen extractable P content progressively increased with the incubation duration in both inoculated and control treatments, varying over 56 days of incubation between 6.84 and 9.04 mg kg^−1^ and 6.84 and 11.06 mg kg^−1^ for control and PSB-inoculated treatments, respectively. The net increase in P observed was 2.20 mg kg^−1^ for the control and 4.22 mg kg^−1^ for PSB-amended treatments at 56 days.

**Table 2:** Mean comparison of main effect on P release (mg kg^−1^) and PSB population (at 56 day) in soil as influenced by P sources and PSBs inoculation at different soil lime content.

Inoculation	Olsen extractable Phosphorus (mg kg^−1^)						
	Days						
	0	7	14	28	56	Net increase in Olsen P over incubation	PSB population at day 56
Phosphorus release (mg kg^−1^) (10^5^ CFU g^−1^ dry soil)							
Control	6.84	7.20	7.78	8.46	9.04	2.20	7.65
PSBs	6.84	7.64	8.52	10	11.06	4.22	8.33
LSD(0.05)	ns	0.104	0.146	0.134	0.150	0.077	7560
**Phosphorous sources**							
SSP	7.92a	8.06a	8.20b	8.36c	8.42c	0.50d	6.73d
Rock Phosphate	6.32c	6.80d	7.16c	7.62d	8.02d	1.70c	7.39c
FYM	6.60b	7.32c	8.58a	10.38b	11.58b	4.98b	8.79b
PM	6.52b	7.54b	8.66a	10.58a	12.22a	5.70a	9.03a
LSD(0.05)	0.114	0.148	0.206	0.188	0.212	0.109	10692
**Lime (%)**							
4.78	7.16a	7.94a	8.90a	10.08a	10.98a	3.82a	8.77a
10	6.94b	7.80a	8.70b	8.78b	10.70b	3.76a	8.48b
15	6.82b	7.50b	8.32c	9.44c	10.28c	3.46c	8.16c
20	6.40c	6.48c	6.66d	7.77d	8.32d	1.92d	6.55d
LSD(0.05)	0.114	0.148	0.206	0.188	0.212	0.109	10692
**Interaction**							
L x PS	*Fig. 1	ns	***Fig. 2	***Fig. 3	***Fig. 4	ns	***Fig. 11
L x I	ns	**Fig. 5	***Fig. 6	***Fig. 7	***Fig. 8	ns	ns
I x PS	ns	ns	ns	***Fig. 9	***Fig. 10	ns	***Fig. 12
L x I x PS	ns	ns	ns	ns	ns	ns	ns
CV	2.88	3.48	4.41	3.54	3.66	11.79	2.32

PS, I, L, ns, ^*,**^ and ^***^indicates phosphorus sources, inoculation, lime, non-significant and significant (LSD test) at p ≤ 0.05, p ≤ 0.01, and p ≤ 0.001 respectively. Means followed by different letter in each column are significantly different at p ≤ 0.05.

**Table 3:** Probability values obtained in three factorial complete randomized design (CRD) for main and interactive effects of PSB, lime and phosphorus sources for P release and PSB population.

Treatments	Day					Net increase	PSB population
	0	7	14	28	56		
PSB	0.958	0.000	0.000	0.000	0.000	0.000	0.000
P sources	0.000	0.000	0.000	0.000	0.000	0.000	0.000
Lime	0.000	0.000	0.000	0.000	0.000	0.000	0.000
Lime x P Sources	*^F1^0.043	0247	***^F2^0.001	***^F3^0.000	***^F4^0.000	0.188	***^F11^0.001
Lime x PSB	0.978	**^F5^0.005	***^F6^0.001	***^F7^0.000	***^F8^0.000	0.992	0.273
PSB x P sources	0.993	0.464	0.184	***^F9^0.000	***^F10^0.000	0.897	***^F12^0.000
PSB x P sources x Lime	0.999	0.830	0.751	0.761	0.056	0.253	0.326

P, PSB, F, ^*,**^ and ^***^indicate phosphorus, phosphate solubilizing bacteria, figure number, significant at probability (P) ≤ 0.05, 0.01 and 0.001 respectively. P values > 0.05 represent non-significant effect upon analysis via three factorial complete randomized design.

It was clear that sources varied significantly in their potential to release P (Tables 2 and 3). Organic sources released more P than did mineral sources. P released over 56 days (in mg kg^−1^) ranged from 7.92–8.42 for single super phosphate (SSP), 6.32–8.02 for rock phosphate (RP), 6.60–11.58 for farm yard manure (FYM) and 6.52–12.22 for poultry manure (PM). The net increase observed in P over 56 days was 0.5, 1.70, 4.98 and 5.70 mg kg^−1^ for SSP, RP, FYM and PM, respectively. During the first 7 days of incubation, SSP released significantly more P into the soil than did other sources, followed by organic sources, while RP produced the lowest Olsen extractable P. In all incubation periods longer than 7 days, organic sources produced or mineralized more P than did mineral sources. At day 14, FYM (8.58 mg kg^−1^) and PM (6.88 mg kg^−1^) mineralized significantly more Olsen extractable P than SSP (8.20 mg kg^−1^), while the lowest magnitude of released P was observed in rock phosphate (7.16 mg kg^−1^). PM mineralized considerably more P than did FYM at day 28 and 56. Similarly, the quantity of P extracted from SSP was significantly higher than RP at day 28 and significantly lower at day 56. The rates of P mineralization/solubilization were 8.9, 30.3, 88.9 and 101.7 µg kg^−1^ day^−1^ over 56 days of incubation for SSP, RP, FYM and PM, respectively. These results showed that SSP is the source with the most readily available P, while FYM, PM and RP are slow-release P fertilizers. Based on the P-release capacity, the sources could be ordered as PM > FYM > RP > SSP. Phosphorus mineralization and solubilization were considerably different in organic and mineral P sources and increased with the passage of time. Initially, SSP released more P than RP or organic sources, but over time the P released from SSP declined and was dominated by organic sources. Among organic P amendments, PM was relatively more efficient than FYM in terms of P release.

Lime application markedly reduced P availability from different P sources in all incubation periods, although as incubation time increased, so did extractable P, as presented in Tables 2 and 3. In general, as the lime content of the soil increased, extractable P was significantly reduced. Soil with naturally occurring lime (4.78%) released significantly more P at all incubation intervals. The lowest P was produced by pots amended with 20% lime (inclusive of naturally occurring) at all incubation intervals. Compared to the initial P content of the soil, the net increases in Olsen extractable P were 3.82, 3.76, 3.46 and 1.92 mg kg^−1^ at 4.78, 10, 15 and 20% lime, respectively. Our finding suggests that lime application reduced Olsen extractable P content in the soil during all incubation intervals.

**Figure 1:**
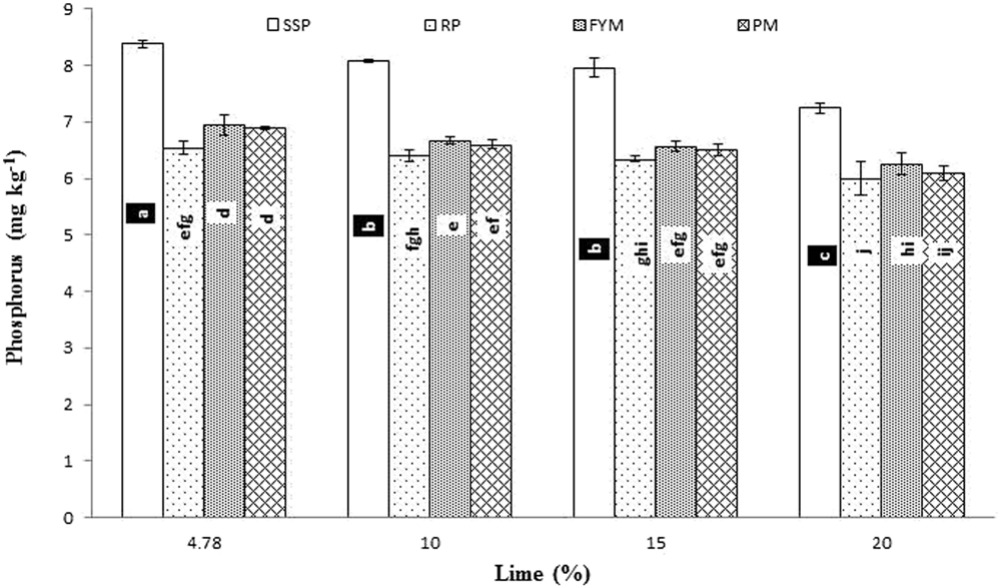
Interactive effect of lime and P sources on soil Olsen extractable P (mg kg^−1^) at day zero. Bars with the different letters are significantly different (*P* ≤ 0.05) according to LSD test. Error bars indicate stander error (n = 3). SSP, RP, PM and FYM indicate single super phosphate, rock phosphate, poultry manure and farm yard manure respectively.

**Figure 2:**
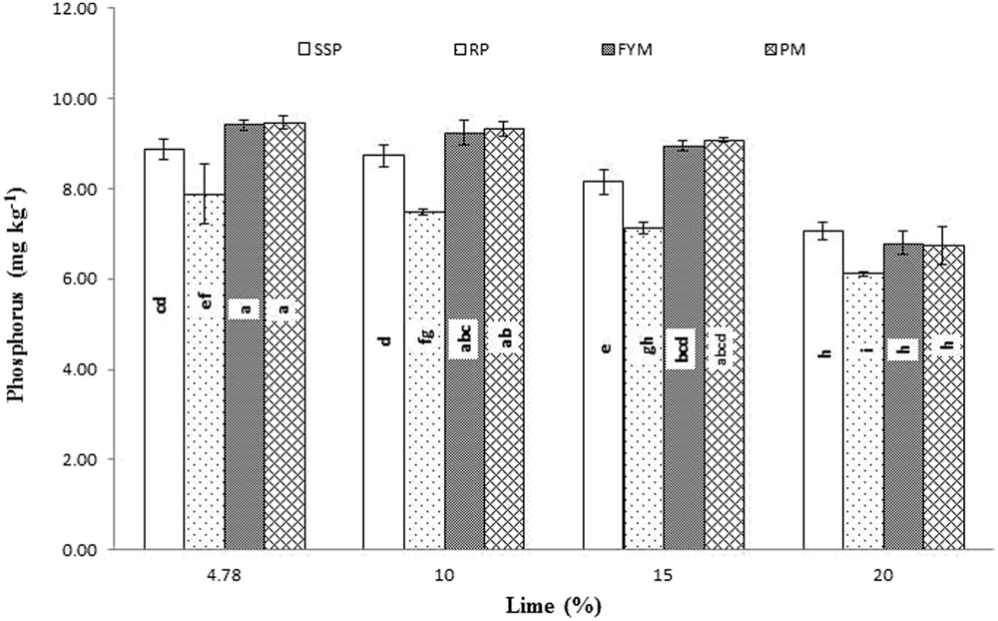
Interactive effect of lime and P sources on soil Olsen extractable P (mg kg^−1^) at day 14. Bars with the different letters are significantly different (*P* ≤ 0.001) according to LSD test. Error bars indicate stander error (n = 3). SSP, RP, PM and FYM indicate single super phosphate, rock phosphate, poultry manure and farm yard manure respectively.

**Figure 3:**
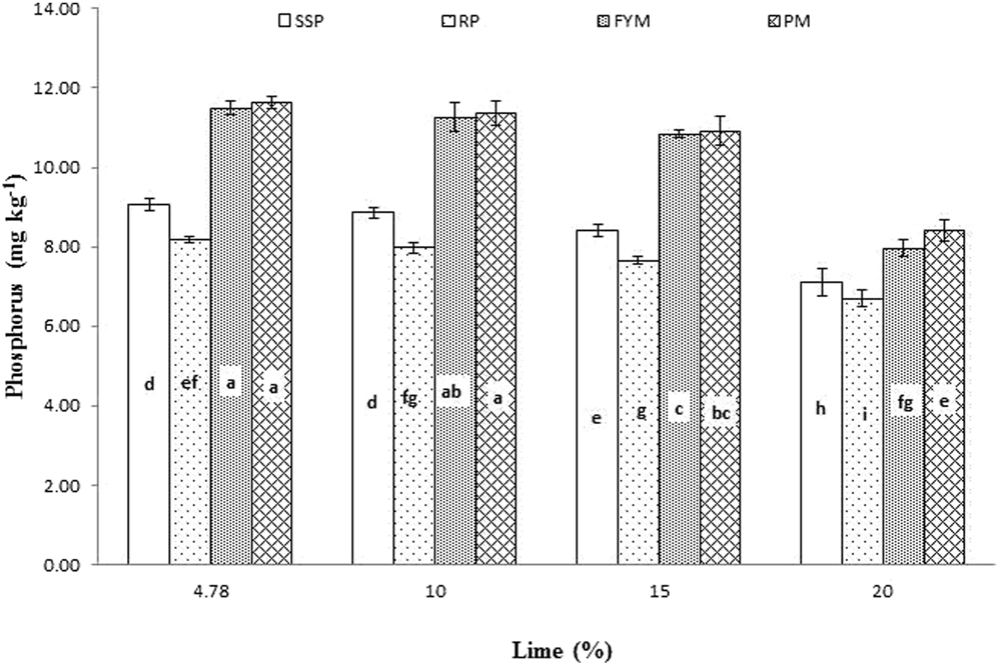
Interactive effect of lime and P sources on soil Olsen extractable P (mg kg^−1^) at day 28. Bars with the different letters are significantly different (*P* ≤ 0.001) according to LSD test. Error bars indicate stander error (n = 3). SSP, RP, PM and FYM indicate single super phosphate, rock phosphate, poultry manure and farm yard manure respectively.

**Figure 4:**
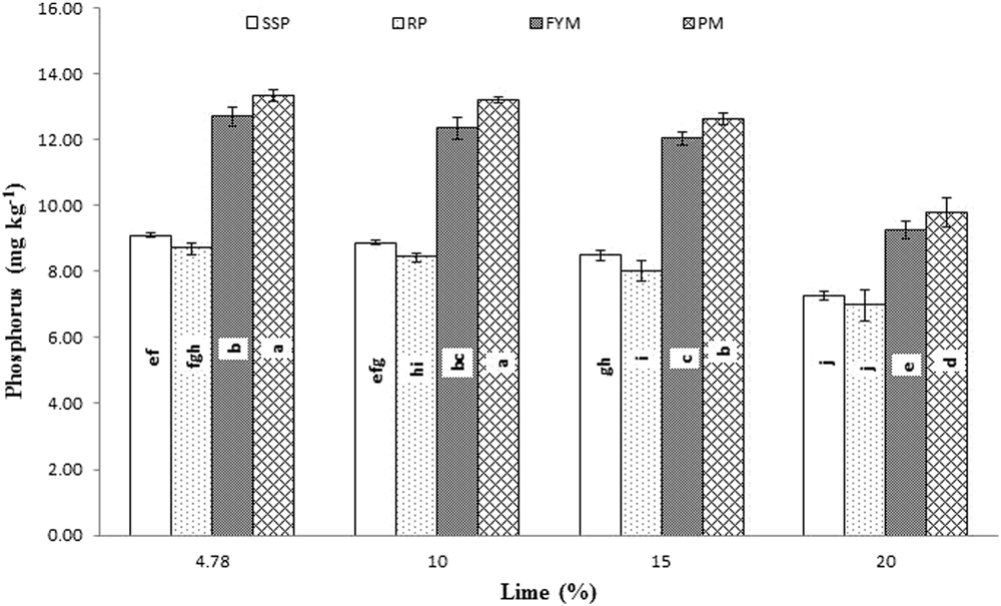
Interactive effect of lime and P sources on soil Olsen extractable P (mg kg^−1^) at day 56. Bars with the different letters are significantly different (*P* ≤ 0.001) according to LSD test. Error bars indicate stander error (n = 3). SSP, RP, PM and FYM indicate single super phosphate, rock phosphate, poultry manure and farm yard manure respectively.

Significant interaction was found between lime and P sources at days 0, 14, 28 and 56 (Figs 1, 2, 3 and 4; Table 3). Initially, SSP released more P, but after longer incubation, organic sources released more P. P release gradually increased with time. A statistically greater amount of P was solubilized from SSP compared to other P sources, but P solubilization from SSP was reduced with added lime on day zero. More P was mineralized from FYM and PM than from RP regardless of lime content. Our findings showed that organic sources mineralized more P than SSP and RP regardless of lime content. Mineralized P decreased with increasing lime content, with the most noticeable decrease occurring at 20% lime. The smallest amount of P was extracted from RP at 20% lime (Fig. 4). Similarly, at day 28, PM and FYM had equivalent effects on soil P content at 4.78, 10 and 15% lime, but both sources released less P at 15% than at 4.78 or 10% lime. At 20% lime, less P was released from all sources. However, PM produced a statistically greater amount of P than RP at all lime levels other than the control and produced an amount equal to that produced by RP at 4.78% lime and to SSP at 15% lime, as presented in Fig. 6. The largest P release was recorded for PM at 4.78 and 10% lime on day 56, and it was significantly greater than FYM at 4.78 and 10% lime and PM at 15% lime. PM with 20% lime released a statistically greater amount of P than did SSP and RP at all lime levels (Fig. 9). Based on these findings, PM could be the best P source for calcareous soils.

**Figure 5:**
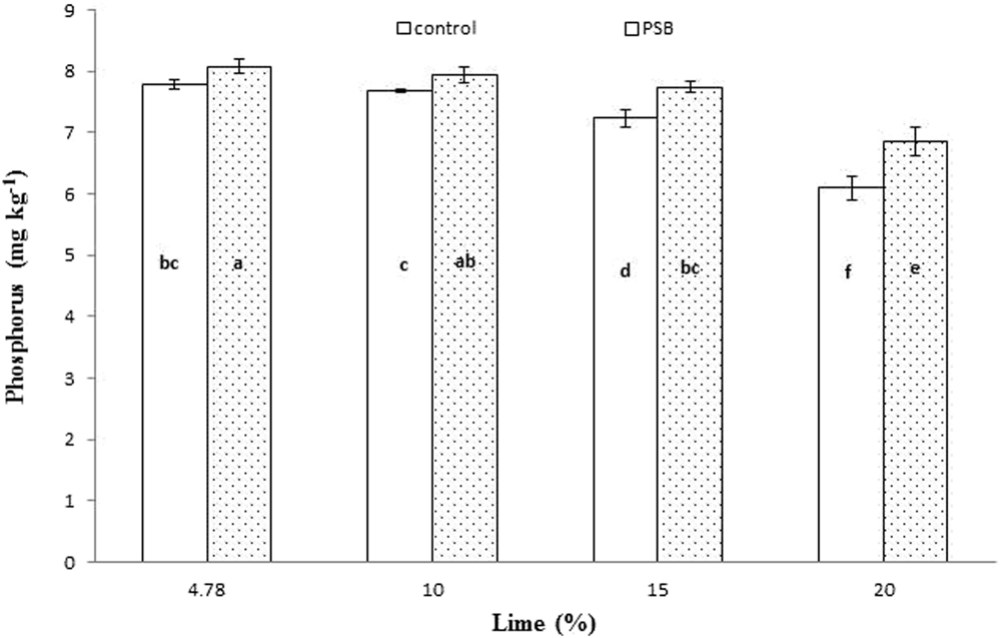
Interactive effect of lime and inoculation on soil Olsen extractable P (mg kg^−1^) at day 7. Bars with the different letters are significantly different (*P* ≤ 0.01) according to LSD test. Error bars indicate stander error (n = 3).

**Figure 6:**
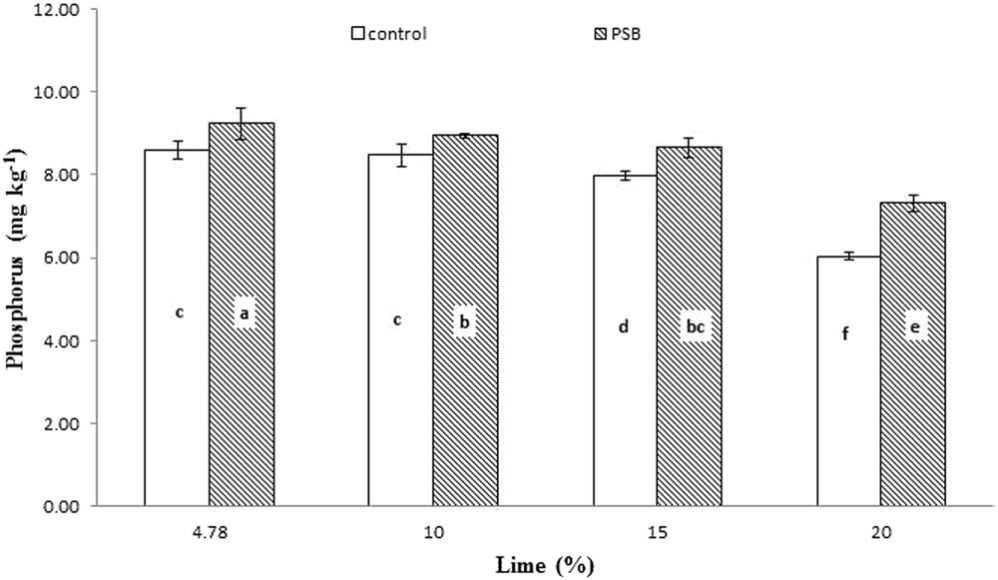
Interactive effect of lime and inoculation on soil Olsen extractable P (mg kg^−1^) in at day 14. Bars with the different letters are significantly different (*P* ≤ 0.01) according to LSD test. Error bars indicate stander error (n = 3).

**Figure 7:**
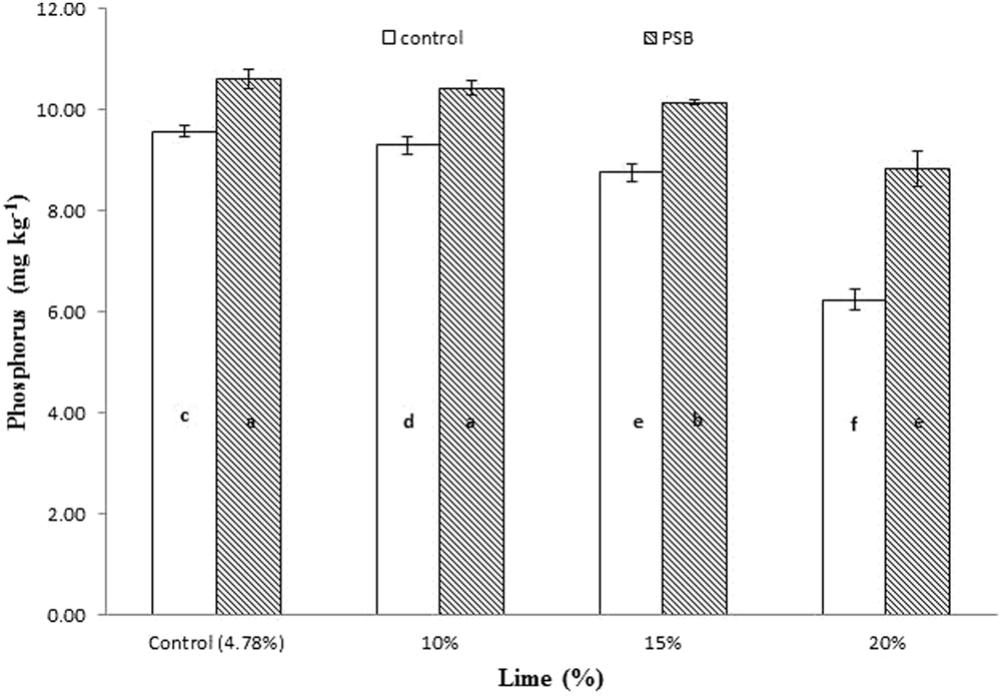
Interactive effect of lime and PSB on soil Olsen extractable P (mg kg^−1^) at day 28. Bars with the different letters are significantly different (*P* ≤ 0.001) according to LSD test. Error bars indicate stander error (n = 3).

**Figure 8:**
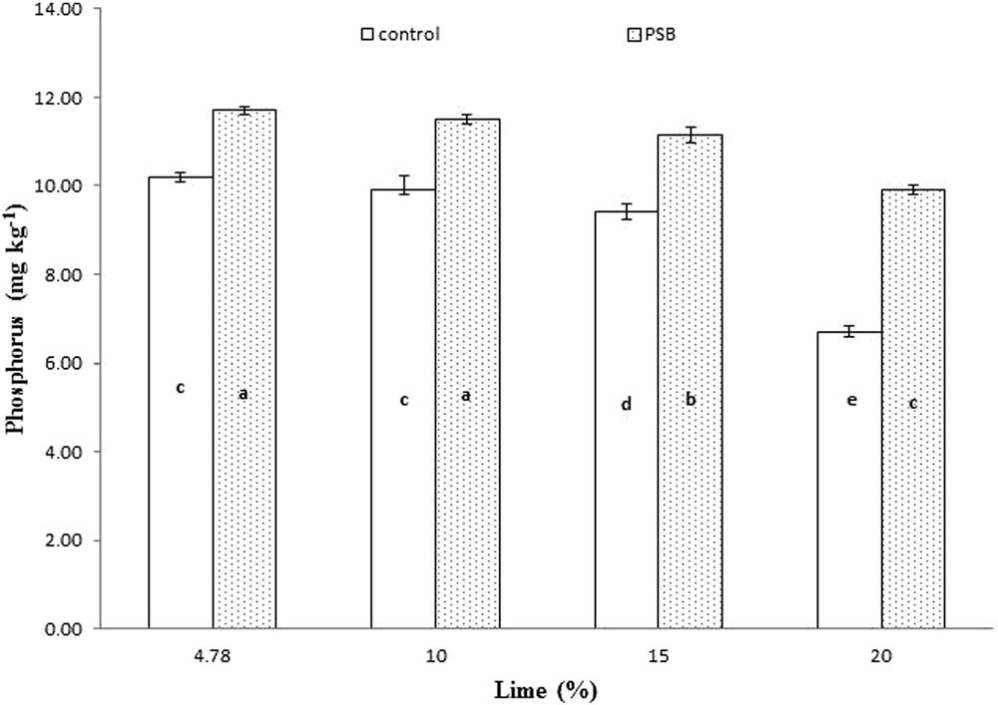
Interactive effect of PSB and lime on soil Olsen extractable P (mg kg^−1^) at day 56. Bars with the different letters are significantly different (*P* ≤ 0.001) according to LSD test. Error bars indicate stander error (n = 3).

Significantly greater interactive responses were noted for lime and PSB on days 7, 14, 28 and 56 (Figs 5, 6, 7 and 8; Table 3). In general, PSB inoculation boosted extractable P at all levels of lime content compared to uninoculated treatments, although in all treatments, as lime content increased, P content substantially decreased, progressively increasing with the duration of incubation. PSB inoculation was found to be useful in counteracting the impact of lime up to 15% in most cases. Pots treated with 10 and 15% lime inoculated with PSB produced statistically equivalent amounts of P as pots treated with 4.78% lime without PSB inoculation. At day 7, treatments amended with 15% lime inoculated with PSB responded like both the control (4.78% lime) and 10% lime without inoculation (Fig. 2). At day 14, 4.78% lime with PSB inoculation had a concentrated amount of P that decreased with increasing lime application. However, a statistically equivalent amount of P existed in 4.78 and 10% lime without PSB inoculation as in 15% lime with PSB inoculation. The lowest P content was observed under 20% lime without PSB inoculation (Fig. 3). Likewise, at day 28, the control and 10% lime with PSB released the most P, while 20% lime without PSB inoculation released the least P. PSB inoculation with 15% lime released more P than the uninoculated control (Fig. 5). Interaction between lime and PSB at day 56 showed that 20% lime with PSB released significantly more P than 4.78, 10 and 15% lime alone. However, 4.78 and 10% lime alone released the same amount of P, which was significantly greater than that released under 15% lime alone (Fig. 8). In our experiment, we observed that PSB were effective at reducing the antagonistic effect of lime on phosphorous mineralization.

**Figure 9:**
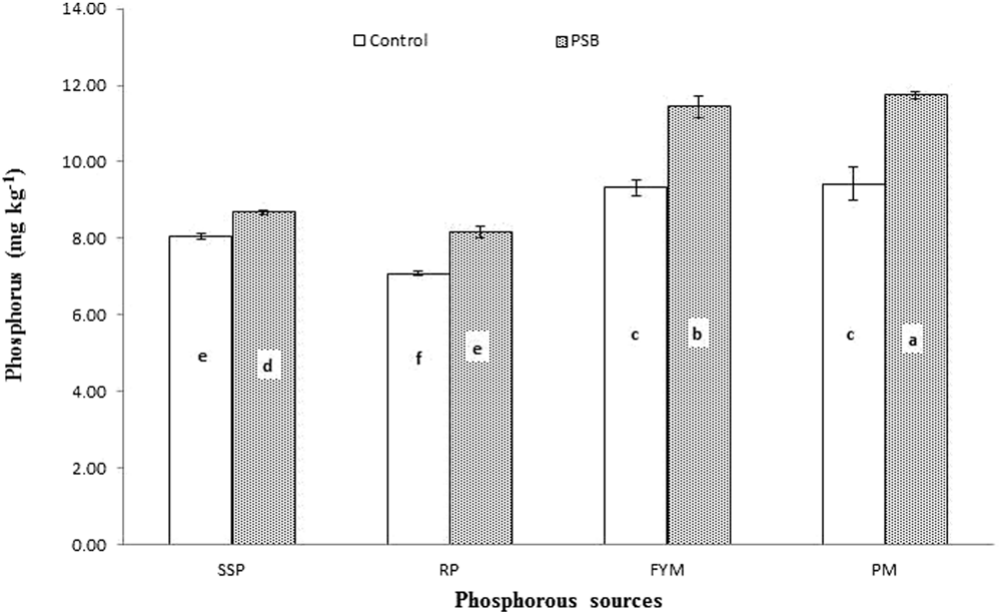
Interactive effect of PSB and P sources on soil Olsen extractable P (mg kg^−1^) at day 28. Bars with the different letters are significantly different (*P* ≤ 0.001) according to LSD test. Error bars indicate stander error (n = 3). SSP, RP, PM and FYM indicate single super phosphate, rock phosphate, poultry manure and farm yard manure respectively.

**Figure 10:**
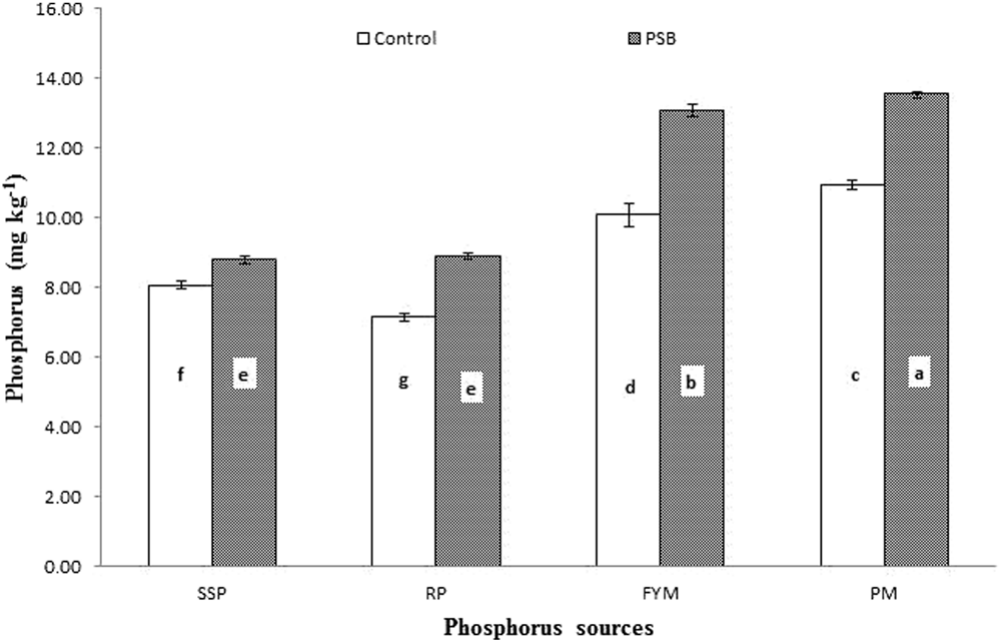
Interactive effect of P sources and PSB on soil Olsen extractable P (mg kg^−1^) in soil at day 56. Bars with the different letters are significantly different (*P* ≤ 0.001) according to LSD test. Error bars indicate stander error (n = 3).

Phosphorus availability showed a response to the integrated effect of PSB inoculation applied with different P sources, and this response was significant at day 28 and 56 (Figs 9 and 10: Table 3), although not significant at any other incubation period. In general, PSB application improved P released from all sources, but at both day 28 and 56 the greatest increase in P released was observed from PM, followed by FYM, SSP and RP. RP applied with PSB released P equivalent to SSP alone. PM and FYM without PSB produced statistically equal amounts of P, which were higher than SSP alone. The least P was released from RP when applied alone at day 28 (Fig. 7). On the 56th day of incubation, PM with PSB mineralized the most P, followed by FYM with PSB. The least P was solubilized from RP with PSB, which was statistically similar to SSP applied with PSB. Similarly, in the absence of PSB, the different sources ordered by the amount of P released were PM > FYM > SSP > RP, as presented in Fig. 10. Different sources varied in their capacity to release P during different incubation periods.

### Post-incubation PSB survival

**Figure 11:**
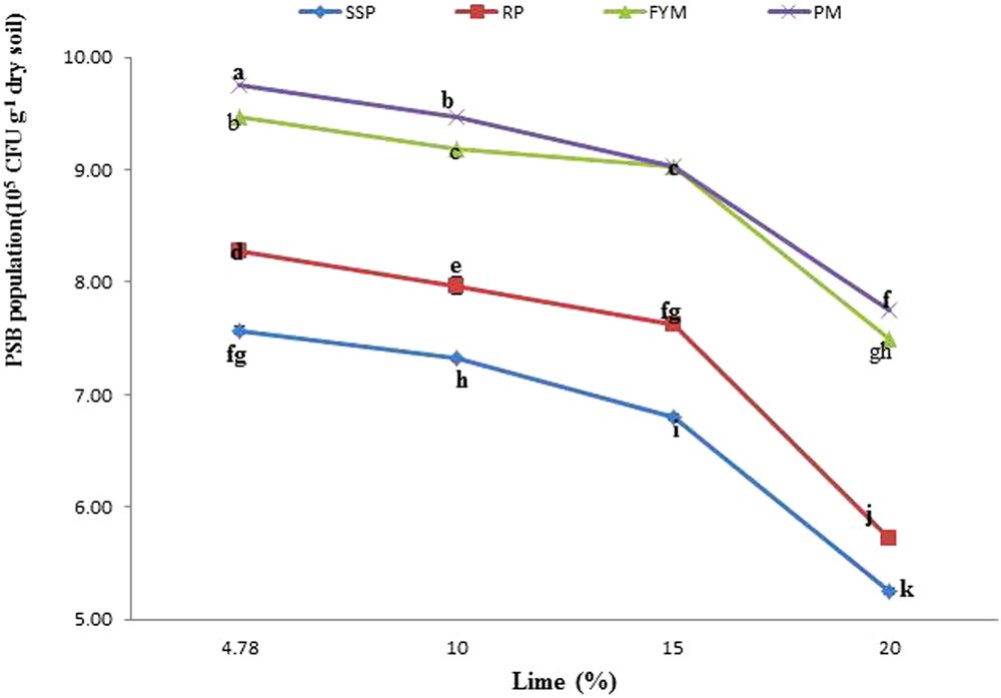
Interactive effect of P sources and lime on PSB population (10^5^ CFU g^−1^ dry soil) in soil at day 56. Lines with the different letters are significantly different (*P* ≤ 0.001) according to LSD test. Error bars indicate stander error (n = 3).

**Figure 12:**
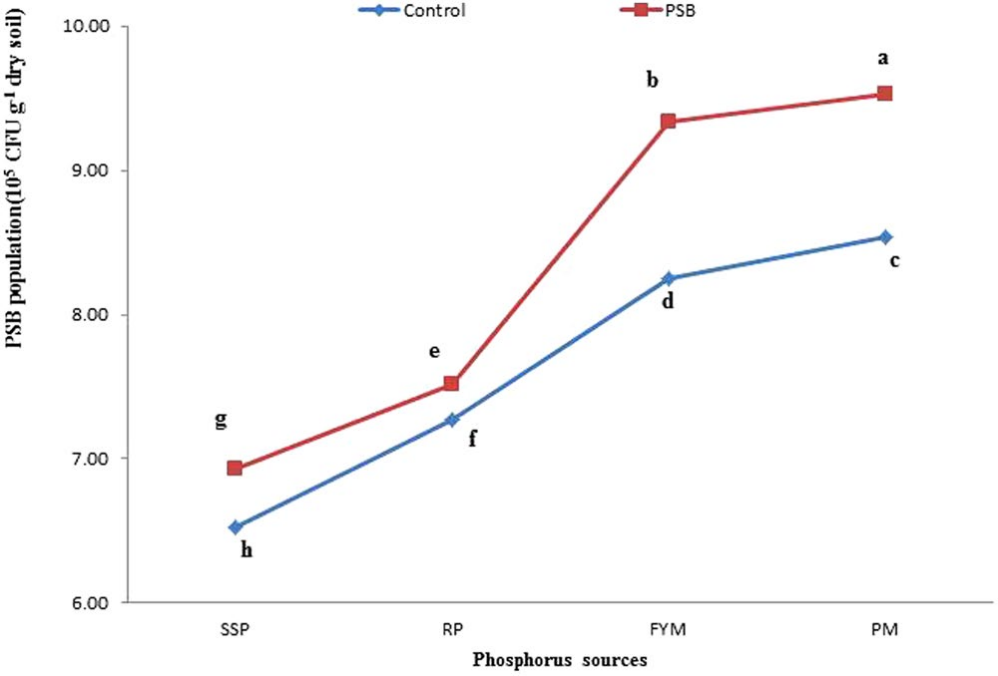
Interactive effect of PSB and P sources on PSB population (10^5^ CFU g^−1^ dry soil) in soil at day 56. Lines with the different letters are significantly different (*P* ≤ 0.001) according to LSD test. Error bars indicate stander error (n = 3).

Analysis of variance revealed that PSB inoculation, P sources and lime content significantly affected the post-incubation PSB population (Tables 2 and 3). The PSB population was significantly larger (8.33 × 10^5^ CFU g^−1^ dry soil) in the inoculated treatments than in the uninoculated treatments (7.65 × 10^5^ CFU g^−1^ dry soil). Among the P sources, PM ranked first in PSB survival, followed by FYM. Rock phosphate produced 7.39 × 10^5^ CFU g^−1^ dry soil, which was significantly lower than organic sources but higher than SSP. Similarly, with the increase in lime content, PSB populations decreased considerably, reaching their minimum at 20% lime. The interactive effects of lime x P sources (L x PS) and inoculation x P sources (I x PS) on post-incubation PSB populations were also very substantial, as presented in Figs 11 and 12, respectively. It was evident from I x PS that with an increase in lime content, the PSB population declined. However, the PSB population decreased more in mineral sources of P than in organic sources. Abundant PSB were observed in PM at 4.78% lime, while PSB were scarce at 20% lime in SSP. Organic sources neutralized the negative effect of lime on PSB populations. PSB in PM and FYM with 20% lime flourished, with statistically equivalent populations to PSB in RP + 15% lime and SSP + 4.78% lime. Those PSB populations were significantly greater than in SSP at any lime level or RP + 20% lime (Fig. 11). Likewise, PSB were more abundant in inoculated samples compared to uninoculated samples. Sources were ranked in the order of PSB population as PM > FYM > RP > SSP in both inoculated and uninoculated samples. Organic sources were proven to be better than mineral sources for PSB populations. PM + PSB propagated the largest PSB population, which was statistically larger than the PSB population in FYM + PSB, followed by that in the PM + control (without PSB) and then in the FYM + control (without PSB). The smallest SPB population was observed in SSP in the uninoculated samples. Among the organic sources, PM was found to be better than FYM for PSB populations, while in mineral sources, RP was better than SSP (Fig. 12). Our findings indicate that PSB may remain viable in the soil for up to 56 days.

## Discussion

### PGPR characteristics of applied PSB inoculum

PSB not only increased P availability in the soil but also performed as plant-growth-promoting bacteria. Khasa *et al*.^21^ stated that PSB improve N, P and K nutrition and may function as biocontrol agents of phytopathogenic fungi, synthesizing phytohormones in the rhizosphere, and as a result may promote plant growth and development. PSB play a vital role in P availability from both organic and mineral sources^22^. This role is attributed to the ability of PSB to produce low-molecular-weight acids^23,24^, such as formic, acetic, propionic, lactic, glycolic, fumaric and succinic acid^25^, which use their carboxyl and hydroxyl groups to chelate cations such as Ca^+2^ and Mg^+2^. This chelation solubilizes insoluble soil phosphorus^13^. According to Bhattarai & Mandal^26^, PSB produce phosphatase, mineral acids and siderophores, all of which increase P solubilization. Chelation, acidification and exchange reactions are primarily responsible for P release from insoluble sources by PSB^27^. Pantoea and Burkholderia species synthesize phosphatase and mineralize phytate^28^. Many scientists have verified the beneficial effect of soil bacteria and fungi on mineral phosphate solubilization from various P sources, including calcium phosphate tribasic [Ca_3_(PO_4_)_2_]^29^, iron phosphate (FePO_4_)^30^ and aluminium phosphate (AlPO_4_). PSB decrease soil pH^31^ by releasing organic acids such as gluconic and keto gluconic acids^32^. Biotic production of protons/bicarbonate release (anion/cation balance) and gaseous (O_2_/CO_2_) exchanges that positively correlate with P solubilization and acid phosphatases produced by PSB have key roles in the mineralization of organic P^33^.

### Phosphorus availability in response to three factors and their interactions: PSB inoculation, P source, and soil calcification

Cumulative P increased due to further addition of the insoluble or immobile P into the mobile pool by rapid mineralization/solubilization from organic matter through acidifying and chelating mechanisms^34^. Mehta *et al*.^35^ found a decrease over time in bioavailable P from soluble P sources due to its precipitation reaction with highly reactive Ca^+2^ ions^36^. This finding also supports our result, as in our case the amount of P released from SSP was initially greater and then fell below that released by PM and FYM as time passed. RP produced or solubilized the least P at all incubation intervals, showing that RP is the least efficient source in alkaline soil. This finding contrasts with the values reported for North Carolina and Syrian RP applied to an acidic Lily soil that showed P dissolutions of approximately 27% after 126 days of incubation. However, the observed values are in the range reported for Indian RP applied under alkaline conditions^37^. Mohammadi *et al*.^38^ reported an ineffective response of soil P to direct application of RP in the short term. Similarly, Saleem *et al*.^39^ found that the addition of RP alone to alkaline soil (pH 7.9) had no significant effect on soil P content.

A substantial quantity of applied P becomes unavailable to plants through complexation under both acidic soil conditions with highly reactive Al^3+^ and Fe^3+^ and calcareous soil conditions with highly reactive Ca^2+^ ^40^. It has also been documented that P anions are very reactive, forming metal complexes with metal cations such as calcium in calcareous soil^41^ and aluminium and iron in acidic soil^42^. These reactions reduce the efficiency of applied P fertilizers by approximately 80%^43^. The decrease in Olsen extractable P due to liming of alkaline soil may be attributed to further increase in soil pH, which tends to decrease Olsen P from 3 to 7 mg kg^−1^ per unit increase in pH, as observed by Shen *et al*.^44^. Liming can also increase phosphate precipitation, which contributes to immobility of P in the soil^45^. Calcification of alkaline soil may boost Ca toxicity, which may further speed up the process of Ca and P bonding/precipitation and thus disturb soil P nutrition.

PM and FYM reduced soil alkalinity by releasing H^+^ ions into the soil^46^ and consequently increased the solubility/mineralization of P from added amendments as well as from naturally occurring P sources. This phenomenon agrees with our findings. Sato *et al*.^8^ observed that long-term application of organic manures results in the formation of soluble Ca–P such as monetite and brushite but no formation of the most stable Ca–P such as hydroxyapatite (HAP). This lack of stable Ca-P formation could be due to the presence of organic anions (i.e., humic, fulvic, tannic and citric acids) that delay the crystallization and transformations of stable Ca–P^8,47,48^ and thus increase P availability in soil. Our findings are also in agreement with those documented by many scientists^49–54^ who reported that PSB release organic acids, lowering the pH of the surrounding soil and thereby solubilizing the fixed calcium phosphate in alkaline soil. Organic acids use their hydroxyl and carboxyl groups to chelate the cations (Ca, Al, Fe) bound to phosphorus and release the P^55^. Alori *et al*.^29^ found that PSB produce the enzyme phosphatase that plays a key role in the solubilization of P. Wu *et al*.^56^ stated that siderophores, chelating compounds and mineral acids synthesized by PSM are also responsible for P solubilization. Similarly, Kumar e*t al*.^57^ reported that PSB produce low-molecular-weight organic acids that acidify the soil and substitute protons for Ca^+2^, solubilizing Ca^+2^-bound P. PSB solubilize insoluble inorganic phosphate compounds, such as tricalcium phosphate, dicalcium phosphate, hydroxyapatite, and rock phosphate^58^.

Begum *et al*.^37^ also observed an increase in P released from different P sources over time. This increase may be attributed to further mineralization of P from organic sources and the important role organic matter plays in P solubilization from mineral sources through acidification and chelation mechanisms. The release of P from mineral sources such as RP and SSP was relatively lower than that from organic sources due to transformation of soluble P into insoluble complexes^35^ as it entered the immobile pools of P through precipitation reactions with highly reactive Ca ions^36^. Among the P sources, RP solubilized the least P into the soil, which is in line with the findings of Saleem *et al*.^39^, who observed a non-significant effect of RP on bioavailable P when applied alone to slightly alkaline soil (pH 7.9) in Faisalabad, Pakistan.

### Post-incubation PSB survival

Our findings indicated that PSB can remain viable in soil for up to 56 days (Table 2), which agrees with the results of a study by Pahari & Mishra^59^, who established that PSBs can survive in soil up to 180 days. Furthermore, Hameeda *et al*.^60^ observed no growth of PSB in uninoculated pots in their study. PSB were more viable in organic sources compared to synthetic and natural P sources, possibly due to secretion of such substances in the decomposition process, which could provide a constant source of nutrition^61,62^ to microbes. Lime reduces bacterial growth in alkaline soil as reported by Six *et al*.^63^, as lime increases soil alkalinity.

## Conclusions

Inoculation with phosphate-solubilizing bacteria (PSB) neutralizes the negative effects of soil calcification on the bioavailability of soil phosphorus (P) from mineral, natural and organic fertilizers. PSB released varying amounts of Olsen extractable P from different P sources during incubation. Soil amended with organic P sources released notably higher Olsen extractable P than SSP and RP in both inoculated and control treatments at all incubation intervals except day zero. PSB viability tended to decrease with increasing lime content; however, organic sources of P better supported PSB growth even with increasing lime content. These findings suggest that PSB increases P availability from organic (FYM and PM), natural (RP) and mineral (SSP) P sources in calcareous and non-calcareous alkaline soils. To improve P nutrition, efficiency and crop growth, P should be supplemented with organic sources in addition to PSB inoculation in alkaline and calcareous soils.

## Methods and Material

### Soil description

This incubation experiment was performed over a period of 56 days, in Soil Microbiology laboratory, at Department of Soil and Environmental Sciences, the University of Agriculture, Peshawar- Pakistan. Our aim was to evaluate the role of phosphate solubilizing bacteria (PSB) in phosphorous (P) availability from organic and inorganic P sources at varying soil lime contents. A surface layer (0–20 cm) of non-calcareous soil (4.78% lime) was collected from cultivated irrigated field, located at 34° 7′ 12″ North and 72° 28′ 20″ East (Gulyana soil series) under wheat-maize cultivation at Agricultural Research Station Swabi, Baja Bamkhel, Distract Swabi, Khyber Pukhtoonkhwa- Pakistan. The physico-chemical attributes of the soil indicated that, it was silty loam in texture, non-saline, non-calcareous, low in organic matter, total N, K and NaHCO_3_ extractable P contents (Table 4).

**Table 4:** Characteristics of soil used for experiment.

Property	Quantity
Bulk density Sand	1.24 gcm^−3^ 26.4%
Silt	67.9%
Clay	5.7%
Textural class	Silt loam
Soil reaction	7.56
Electrical conductivity (EC_e_)	1.76 dSm^−1^
Lime	478 g Kg^−1^
Organic matter content	8.2 g Kg^−1^
Total nitrogen content	0.08 g Kg ^−1^
NaHCO_3_ extractable P	5.28 mg Kg^−1^
Potassium	78 mg Kg^−1^

### Experimentation

The experiment was arranged in three factorial completely randomized design (CRD) with three repeats, consisting of two types of inoculation (control, PSB), four sources of phosphorus [single super phosphate (SSP), rock phosphate (RP), poultry manure (PM) and farm yard manure (FYM)] to obtain 45 mg P_2_O_5_ kg^−1^ soil and four levels of lime (4.78, 10, 15 and 20%), making a total of 32 treatments per repeat. The soil obtained was shade dried, sieved (2 mm) and distributed in 96 plastic incubation pots at the rate of 100 ± 2 g (inclusive of natural lime) per pot with approximately 15% (V/M) moisture. The lime was applied to pots according to the prosed structure 30 days before than P sources and PSB. RP, PM and FYM were analyzed for N, P and K concentrations before application, which contained 0, 22.5,13.4 g N kg^−1^, 170, 13.9, 8.7 g P kg^−1^ and 0, 12.7, 10.2 g K kg^−1^, respectively. Basal dose of nitrogen (N) and potassium (K_2_O) at the rate of 60 and 30 mg kg^−1^ as urea and sulphate of potassium (SOP), respectively, inclusive of N and K obtained from organic sources were applied to all treatments. Mineral fertilizers and sources were applied at desired rate in solution to properly mix it with soil. Calcium carbonate (CaCO_3_) was applied at the rate of 5.22, 10.22 and 15.22 g per pot (100 g soil) for obtaining 10, 15 and 20% of soil lime content, respectively in addition to soil natural lime content. Peat-based inoculum of PSB obtained from National Agriculture Research Center (NARC) containing 1.75 × 10^8^, CFU of PSB g^−1^ inoculum (wet weight), was added at the rate of 1 kg ha^−1^ as 1% (M/V) suspension made in sterile distilled water. Viable cell count of PSB as determined by dilution plat techniques was 1.71 × 10^6^ CFU ml^−1^ in 1% (M/V) prepared suspension, 5 ml of which was added to each pot receiving PSB^64^ and 5 ml of sterilized distilled water were added to control (without PSB) pots. After treating the soil was properly mixed and pots were randomized periodically. The pots were incubated at 32 ± 2 °C. Moisture content of the soil was kept at about 50% of field capacity throughout the experiment on daily basis by weighing the pots and making up the loss of water through addition of sterilized distilled water. Ten gram of soil was taken out at an interval of 0, 7, 14, 28 and 56 days of incubation and analyzed each for Olsen NaHCO3 extractable P and moisture content.

### Composition of PSB inoculum

PSB inoculum was obtained from NARC and analysed for its bacterial composition according to Bergeys manual of systematic bacteriology^65^ and Bergeys manual of determinative bacteriology^66^ and the genus bacillus^67^ on modified Pikovskaya’s agar medium containing insoluble inorganic forms of P like Ca_3_(PO_4_)_2_. The mean population of phosphate solubilizing bacteria (PSB) in the inoculum was 1.75 × 10^8^ CFU of PSB g^−1^ (wet weight). It was observed that inoculum was composed of Pseudomonas (12.7%), Pantoea (10.2%), Mycobacterium (13.6%), Bacillus (15.5%), Rhizobia (9.40%), Burkholderia (10.3%), Arthrobacter (8.50%) and Enterobacter (2.80%) while, 17% of the colonies were unidentifiable. Most of the bacterial species identified were from PSBs when compared with the literature. Bashan *et al*.^68^ found *Pseudo-monas*, *Bacillus*, *Rhizobium* and *Enterobacter* along with *Penicillium* and *Aspergillus* as most powerful P solubilizers. Kumar and Shastri^69^ cited that *B*. *sub-tilis*, *B*. *polymyxa, B*. *sircalmous, Bacillus megaterium*, *B*. *circulans*, *Pseudomonas striata*, and *Enterobacter* could be considered as the most significant strains of PSB. Satyaprakash *et al*.^49^ also declared *Bacillus*, *Pseudomonas, Rhizobium, Aspergillus* and *Penicillium* as effective P solubalizers.

### Data collection

Soil pH and electrical conductivity (EC) were determined by pH and EC meter in 1:2 soil water suspension by the procedure of Thomas^70^ and Rhoades^71^, respectively. N in soil was determined by Kjeldhal method of Mulvany^72^ and extractable potassium by the method of Ryan *et al*.^73^. Phosphorous in soil was measured by Olsen NaHCO_3_ method (Olsen *et al*.)^74^ by extracting samples with 0.5 M, NaHCO_3_ (pH 8.5) at a solution/solid ratio of 20:1 for 30 min. CaCO_3_ content of soil was determined by titration method of Loeppert and Suarez^75^, texture by Ghee and Bauder^76^ and soil organic matter content was measured by method of Nelson and Sommers^77^.

Phosphate solubalization by PSB was quantified by using National Botanical Research Institutes phosphate growth medium (Nautiyal)^78^ and P content in culture supernatant was determined by the para molybdenum blue method (Olsen *et al*.)^74^. Siderophores production by PSB was evaluated by chrome azurol S method described by Alexander and Zuberer^79^. IAA production by PSB was determined on Yeast Extract Mannitol broth medium by the procedure of Vincet^80^. Alkaline phosphatase activity (µg PNP h^_1^ g^_1^ soil) of PSB was determined by the method of Eivazi and Tabatabai^81^. Post incubation PSB population in each pot was also measured in triplicates by suspension dilution plate techniques in fresh soil samples using Pikovskaya’s medium (Pikovskaya)^82^. Total number of phosphate-solubilizing bacterial (PSB) colonies were counted and calculation on CFU were made according to procedure of dilution plate techniques.

### Statistical Analysis

Data collected on P release and post PSB population was analyzed by Fisher’s (F) test for three factorial completely randomized design (CRD) using the method of Steel and Tore^83^ via Statistical Package SAS 9.1 and subjected to least significant difference test (LSD) for comparing the difference between means in case of significant F test. Data regarding PGPR characteristics was analyzed by descriptive statistics.
