# Author Correction: Phosphate-Solubilizing Bacteria Nullify the Antagonistic Effect of Soil Calcification on Bioavailability of Phosphorus in Alkaline Soils

**DOI:** 10.1038/s41598-018-22653-7

**Published:** 2018-03-07

**Authors:** Muhammad Adnan, Zahir Shah, Shah Fahad, Muhamamd Arif, Mukhtar Alam, Imtiaz Ali Khan, Ishaq Ahmad Mian, Abdul Basir, Hidayat Ullah, Muhammad Arshad, Inayat-Ur Rahman, Shah Saud, Muhammad Zahid Ihsan, Yousaf Jamal, Amanullah, Hafiz Mohkum Hammad, Wajid Nasim

**Affiliations:** 1 Department of Agriculture, The University of Swabi, Swabi, Pakistan; 2 Department of Soil and Environmental Sciences, the University of Agriculture, Peshawar, Pakistan; 3 College of Plant Science and Technology, Huazhong Agricultural University, Wuhan, China; 4 Department of Agronomy, the University of Agriculture, Peshawar, Pakistan; 5 Mountain Agriculture Research Center Gilgit Bultistan, Bultistan, Pakistan; 6 College of Horticulture, Northeast Agricultural University, Harbin, China; 7 Cholistan Institute of Desert Studied, The Islamia University of Bahawalpur, Bahawalpur, Pakistan; 8 Deparment of Agronomy, the Islamia University of Bahawalpur, Bahawalpur, Pakistan; 9 Department of Environmental Sciences, COMSATS Institute of Information Technology, Vehari, 61100 Pakistan; 10 CIHEAM-Institute Agronomique Mediterraneen de Montpellier (IAMM), Montpellier, 34090 France; 11 CSIRO Ecosystems Sciences and Sustainable Agriculture Flagship, Toowoomba, QLD 4350 Australia

Correction to: *Scientific Reports* 10.1038/s41598-017-16537-5, published online 23 November 2017

The original version of this Article contained a typographical error in the spelling of the author Inayat-Ur Rahman, which was incorrectly given as Inayat-Ur Rehman. This has now been corrected in the PDF and HTML versions of the Article.

